# Response of Rocky Reef Top Predators (Serranidae: Epinephelinae) in and Around Marine Protected Areas in the Western Mediterranean Sea

**DOI:** 10.1371/journal.pone.0098206

**Published:** 2014-06-06

**Authors:** Carlos Werner Hackradt, José Antonio García-Charton, Mireille Harmelin-Vivien, Ángel Pérez-Ruzafa, Laurence Le Diréach, Just Bayle-Sempere, Eric Charbonnel, Denis Ody, Olga Reñones, Pablo Sanchez-Jerez, Carlos Valle

**Affiliations:** 1 Departamento de Ecología e Hidrología, Universidad de Murcia, Murcia, Spain; 2 Aix-Marseille Université, Université du Sud Toulon-Var, CNRS/INSU/IRD, Institut Méditerranéen d’Océanologie (MIO), Marseille, France; 3 GIS Posidonie, Institut Méditerranéen d’Océanologie (MIO), Marseille, France; 4 Departamento de Ciencias del Mar y Biología Aplicada, Universidad de Alicante, Alicante, Spain; 5 Parc Marin de la Côte Bleue, Observatoire, Carry-le-Rouet, France; 6 WWF-France, Marseille, France; 7 Instituto Español de Oceanografía, Centro Oceanográfico de Baleares, Palma de Mallorca, Spain; Aristotle University of Thessaloniki, Greece

## Abstract

Groupers species are extremely vulnerable to overfishing and many species are threatened worldwide. In recent decades, Mediterranean groupers experienced dramatic population declines. Marine protected areas (MPAs) can protect populations inside their boundaries and provide individuals to adjacent fishing areas through the process of spillover and larval export. This study aims to evaluate the effectiveness of six marine reserves in the Western Mediterranean Sea to protect the populations of three species of grouper, *Epinephelus marginatus*, *Epinephelus costae* and *Mycteroperca rubra*, and to understand in which circumstances MPAs are able to export biomass to neighbouring areas. All the studied MPAs, except one where no grouper was observed, were able to maintain high abundance, biomass and mean weight of groupers. Size classes were more evenly distributed inside than outside MPAs. In two reserves, biomass gradients could be detected through the boundaries of the reserve as an indication of spillover. In some cases, habitat structure appeared to exert a great influence on grouper abundance, biomass and mean individual weight, influencing the gradient shape. Because groupers are generally sedentary animals with a small home range, we suggest that biomass gradients could only occur where groupers attain sufficient abundance inside MPA limits, indicating a strongly density-dependent process.

## Introduction

Several marine species are seriously threatened by an array of anthropogenic actions [1], from which fishing is likely the main human activity seriously affecting fish population abundance and size structure, and causing marine biodiversity loss. Worm et al. [2] stated that although increasing efforts to restore marine ecosystems and rebuild fisheries are under way, most fish stocks worldwide still require rebuilding. Lower exploitation rates are needed to reverse the collapse of vulnerable species, such as high trophic level species, which are likely to cause upheavals in the global ecosystem through the loss of particular functions played by these key species (e.g. reduction of natural predation) [2].

Recent studies, based on meta-analyses and reviews, show that MPAs can reverse most deleterious effects of fisheries on the marine environment [3]–[8] provided that they are properly managed [9], [10]. The notable effects of marine reserves are an increase in abundance and an enlargement of the average size of individuals of the target species inside the boundaries of the protected area, so that greater abundance and size theoretically imply an increase in reproductive potential [3]–[5], [7]–[8]. Eggs and larvae from restored spawning stocks inside MPAs could then be exported by currents to adjacent fishing areas ([11], but see [12]). On the other hand, because of increased density inside the MPA, adults and juveniles fishes from target species may emigrate from inside the protected locations to outside where the density is lower (“spillover”, [13]). An indirect method to estimate the magnitude and importance of such export of larvae, juveniles and adults fishes from MPA to neighbouring areas is to look for the likely existence of gradients of biomass of target species across MPA limits, under the rationale that, if spillover occurs, there would be more fishes near than far away from the MPA [6], [14].

This research strategy has been used in several studies in the Mediterranean (e.g., [15]–[18]) and worldwide (e.g., [19]–[20]). It has been hypothesized that the shape of biomass gradient for a given fish population responds to the distribution of the fishing pressure outside the reserve, and to the flux of individuals over the reserve boundary, which in turn would depend on the extent to which the system’s carrying capacity is reached by the population [6]. The instantaneous population growth rate of a species would affect the speed of recovery of the population after cessation of fishing activity and the ability of the species to maintain abundances close to the carrying capacity inside the integral reserve; even more importantly, growth rate likely determines the fishing mortality that the population can support without collapsing [6]. For its part, flux of adults through MPA limits will depend on movement patterns, home range and spatial use of the species concerned [4], [6], [21].

On the other hand, habitat structure is one of the factors explaining the small-scale spatial variability of fish assemblage [22]–[23], and spatial variations in habitat structure is likely to affect the strength and even the occurrence of biomass increase within MPA boundaries and spillover [14], [16], [21], [24], by influencing resource availability (food or refuge against predators or fishing), and ultimately affecting population growth and mobility.

Among the most common species affected by fishing pressure are top predators [25]–[27], and especially groupers [28], [29]. Groupers (Perciformes: Serranidae: Epinephelinae) are emblematic species around the world, as they are of great importance for both recreational and artisanal fisheries [30], [31]. Many species of Epinephelinae are overexploited, and about 25% of the species are under some level of threat [31]. The high susceptibility of grouper species to overfishing and habitat loss is likely due to their biology and life style, which promote a synergetic effect with anthropogenic activities. High site fidelity, high longevity, late maturity, formation of spawning aggregations, slow growth rate and low resilience (5 to 14 years to minimum population doubling time) are some of the characteristics that determine a high to very high level of vulnerability of these species [31].

The aim of this study is to evaluate the efficiency of protection measures to promote the recovery of populations of three groupers species both within and around MPAs in the Western Mediterranean Sea. The hypotheses to be tested are whether, and to what extent, there are higher grouper abundances inside than outside marine reserves, and whether biomass gradients can be found across the boundaries of the studied MPAs, suggesting spillover to neighbouring areas. Moreover, the present study aims at exploring the possible interference of the spatial distribution of structural habitat on grouper abundance within MPAs, and the occurrence of spillover towards adjacent areas.

## Materials and Methods

Data acquisition for this work was made by visual censuses only; no animals were collected or manipulated. Research permissions were provided by “Ministerio de Medio Ambiente, Medio Rural y Marino”, “Servicio de Pesca y Acuicultura – Comunidad Autónoma de la Región de Murcia”, and “Departament de Medi Ambient y Habitatge – Generalitat de Catalunya” for Spanish MPAs, and “Ministère de l’Écologie, du Développement durable et de l’Énergie” for MPAs located in France.

### Study Area

**Figure 1:**
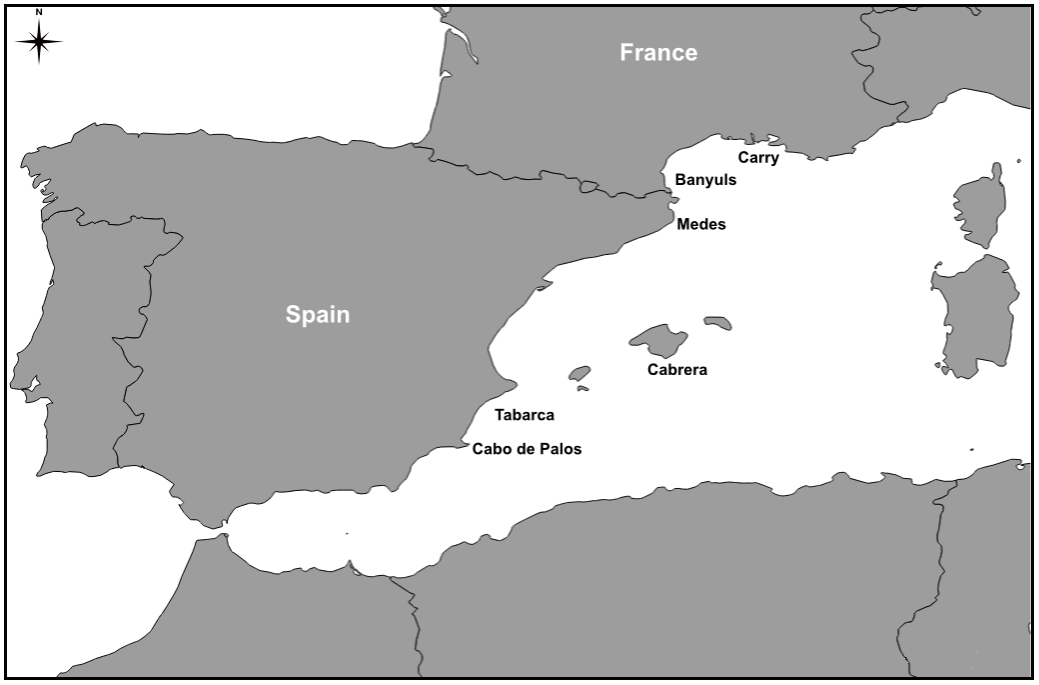
Location of the Mediterranean marine protected areas (MPAs) studied. 1: Carry-le-Rouet, 2: Banyuls, 3: Medes, 4: Cabrera, 5: Tabarca, 6: Cabo de Palos.

The work was conducted from July to October of 2003 and 2004 in six MPAs spread over the Western Mediterranean Sea (Fig. 1): the natural marine reserve of Cerbère-Banyuls (hereafter called Banyuls) and the Carry-le-Rouet (Carry) marine park in France, and the National Park of Cabrera (Cabrera), and the marine reserves of Medes islands (Medes), Tabarca island (Tabarca) and Cabo de Palos – Hormigas islands (Cabo de Palos) in Spain. All MPAs (except Carry) include one or more no-take zones surrounded by buffer zones, where some uses are permitted (usually recreational diving and some kind of artisanal fishing). Carry, is formed exclusively by a no-take zone, although it belongs to a larger conservation unit in the region. Common criteria used to select the MPAs included in this study were that they were established for more than 10 years, and presented a high level of enforcement. All marine reserves involved in this study are similar regarding the composition and constitution of the seabed, presenting *Posidonia oceanica* (Posidoniaceae) meadows and rocky bottoms.

### Sampling Design and Data Acquisition

Seven to nine sectors, separated by 1000’s of metres, were positioned at increasing distances from the core of each MPA. In each sector, three zones were haphazardly located at a scale of 100s of metres. Finally, six transects (replicates) separated by 10s of metres were sampled in each zone. In three MPAs (Banyuls, Carry and Cabo de Palos), gradients in fish parameters were studied along three sectors located inside the MPA (one inside the no-take zone and two within the buffer zone (herafter NTZ and BZ respectively) and six sectors in unprotected areas (UP), where fishing is allowed: three in one direction and three in the opposite direction (Table 1). In the three MPAs located on islands (Cabrera, Medes and Tabarca) only one direction (northward) could be studied due to the absence of suitable rocky habitats southward (Table 1). In Cabrera, three sectors were located within the NTZs, three sectors inside the BZs, and three within UP, outside the MPA. For Medes, three sectors were located within the NTZ, one in the BZ, and three outside the MPA (Table 1). In Tabarca, the sampling was performed within: one sector inside the NTZ, three sectors in the BZ, and the rest outside the reserve (Table 1). In this MPA, the sampling was done on two different bottom types: rocky bottoms and *Posidonia oceanica* meadows, respectively named Tabarca – rocky and Tabarca - *Posidonia* hereafter (see Table 1 in Harmelin-Vivien et al. [16] for further information).

**Table 1:** Summary of spatial sampling effort applied in each studied MPAs, depending on MPA management plan and natural habitat distribution, showing the number of sectors sampled in each existing level of protection.

MPAs	Sectors			Zones	Transects
	NTZ	BZ	UP		
Marine Park of Carry-le-Rouet	3	0	6*	27	162
Natural Marine Reserve of Cerbère-Banyuls	1	2	6*	27	162
Marine Reserve of Medes Island	3	1	3†	21	126
National Park of Cabrera Island	3	3	3†	27	162
Marine Reserve of Tabarca Island	3	1	3†	21	126
Marine Reserve of Cabo de Palos-Hormigas Islands	1	2	6*	27	162

In each sector, 3 random zones and 6 replicas were always defined in each MPA. NTZ: No-take zone; BZ: Buffer zone; UP: unprotected area; Zones: total number of zones randomly located; Transects: total number of replicates.

*Three sectors were located to the South and other 3 to the North.

† All sectors located to the North direction.

Three species of groupers were assessed during this study, *Epinephelus marginatus* (Lowe, 1834) (dusky grouper), *E. costae* (Steindachner, 1878) (goldbloch grouper) and *Mycteroperca rubra* (Bloch, 1793) (mottled grouper). According to IUCN red list, *E. marginatus* is defined as an “endangered” species, while *E. costae* is categorized as “data deficient” and *M. rubra* as “least concern”. The abundance of these species was assessed by visual census in 25×5 m transect belts located at 6–12 m depth and parallel to the coast. As *P. oceanica* beds covered large areas around Tabarca island, seagrass beds were surveyed in this habitat in 50×5 m transects, as fishes were more dispersed. In each transect, fishes were identified and the size of each individual was recorded within 10-cm size classes, so that fish weight could be estimated from length-weight relationship found in the literature. Within each transect the following descriptors of structural habitat were also registered: number of rocky boulders (classified as small, medium and large) and percentage of cover by different types of substrate (namely rock, sand, *Posidonia* and pebbles), for further details on habitat data acquisition, see García-Charton et al. [24] and Harmelin-Vivien et al. [16].

### Data Analysis

The effect of protection on groupers was evaluated separately for each MPA, because differences in sampling design precluded making a unique analysis with all MPAs together. Distance-based permutational multivariate analysis of variance (PERMANOVA, [32]) based on Bray-Curtis dissimilarities [33] on log-transformed data was run in univariate mode, using abundance, biomass and individual mean weight of the three target species of groupers as response variables. In general, the field experimental design for each MPA consisted of three factors: Location (factor L, fixed, comparing two or three levels of protection in each MPA, depending on the MPA, considering NTZ and BZ as compared to the unprotected –UP– locations), Sector (factor S, 3 levels, random, nested in L) and Zone (factor Z, 3 levels, random, nested in S). Only in the case of Cabrera all NTZ, BZ and UP levels were compared. For Medes and Tabarca, one sector (in BZ) was excluded from the analyses to get a balanced design, so that only the comparison between NTZ and UP was taken into account. In the case of MPAs where 6 sectors were surveyed outside the protected location against 3 sectors within it (Carry, Banyuls and Cabo de Palos), an asymmetrical design was considered, for which the Location term was partitioned into two portions: the one degree-of-freedom contrast Protected (P) (including NTZ and BZ locations) *vs*. UPs locations, and the variability between UPs. The overall mean squares of the terms S(L) and Z(S(L)) were similarly partitioned into S(P *vs* UPs) and S(UPs), and Z(S(P *vs* UPs)) and Z(S(UPs)), respectively. Nevertheless, as no groupers were recorded in Carry, and in Banyuls they were observed exclusively in the NTZ (see results), only the case of Cabo de Palos was explored using this asymmetrical design. In order to minimize the effect of habitat variability on data and exploring only the effect on species protection, we used the environmental data as covariables. For all analysis 9999 permutations were applied under a full model, using PRIMER v.6 package.

Trends and significance of gradients of grouper biomass across MPAs boundaries were calculated using linear correlation with the distance from MPA limits at the scale of zones. Negative correlations would indicate that biomass decreases from the core of MPA to distant fished zones. The border of the NTZ was defined as zero, so negative distances indicate zones inside the NTZ, and positive distances zones outside the NTZ. To explore the actual shape of grouper biomass gradients across MPA borders, generalized additive modelling (GAM) were applied using gam v.1.06.2 in R statistic package. GAM is known to be useful when the actual relationship between the variables is unknown and expected to be of a complex form, not easily fitted by standard linear or non-linear models [34]. Distance to the boundary of the no-take zone was introduced as a continuous smooth variable modelled non-parametrically using a loess smoother (lo(Distance)). We applied to model Gaussian variance and identity link functions, both based on Hastie and Tibshirani [34] and Venables and Ripley [35]. The gradients were tested for both sides of MPA (South and North), or as a unique gradient, depending on the study case.

**Figure 2:**
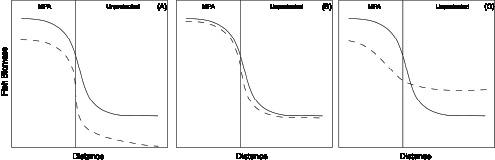
Hypotheses for the shape of gradient of fish biomass across MPA boundaries with raw data (solid line) and residual data after extracting habitat influence (dotted line) where habitat quality (a) is equally good or (b) has no influence both inside and outside the MPA, and (c) where habitat quality is better within the MPA. The vertical line indicates the limit of the MPA, with protected area to the left and fishes zones to the right.

The likely influence of habitat structure on gradient shape was further assessed by performing multiple linear regression analyses to measure the strength of the relationship between the whole set of habitat variables (including their quadratic and cubic terms) and each species’ population parameter (abundance, biomass and individual mean weight). Prior to analyses, the extreme and influential cases were detected and removed by carrying out analysis of residuals [36]–[37]. Then, the residuals of these analyses were used as a dependent variable in linear correlations and GAMs with the distance from MPA limits [16]. If habitat quality is equal both inside and outside the MPA, or does not influence fish biomass, we hypothesize that the extraction of habitat influence would not affect the shape of biomass gradient as depicted by GAMs (Fig. 2a and b). If habitat quality is better inside than outside the MPA (i.e. it promotes higher fish biomass within the MPA because structural habitat provides either enhanced food or/and refuge resources as compared to surrounding areas), the shape of biomass gradient across MPA limits would be smoothed once extracted the influence of habitat from raw data (Fig. 2c).

## Results

### Effects of Protection on Grouper Abundance and Size Structure

**Figure 3:**
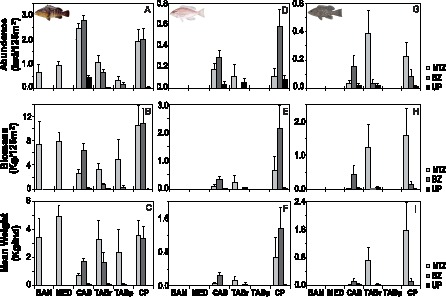
Abundance, biomass and mean weight of the three species of groupers, *E. marginatus*, *E. costae* and *M. rubra* according to the tree levels of protection (No-take zone: NTZ, Buffer zone: BZ and unprotected: UP) in all MPAs studied (BAN: Banyuls; MED: Medes; CAB: Cabrera; TAB-r: Tabarca Rocky habitat; TAB-p: Tabarca *Posidonia* habitat; CP: Cabo de Palos), Carry excepted as no groupers were recorded there.

*Epinephelus marginatus* was recorded in all MPAs, except Carry. In Banyuls and Medes, this grouper was censused only inside the NTZ, and in Tabarca – *Posidonia* this species was censused both in NTZ and BZ areas, but not outside the MPA. The two other species, *E. costae* and *M. rubra*, were recorded only in Cabrera, Tabarca – rocky and Cabo de Palos MPAs. Thus, the effect of protection levels and nested spatial factors on the abundance and biomass of all grouper species will be explored by PERMANOVA only for the latter three case studies (Fig. 3). Cabrera and Cabo de Palos MPAs presented the highest grouper abundance amongst all reserves, while the lowest abundance values were recorded in the northernmost MPAs where these species were observed (Banyuls and Medes).

Further to the fact that in Banyuls, Medes and Tabarca – *Posidonia E. marginatus* was observed only inside the protected areas, the abundance, biomass and mean weight of this species were significantly higher within the protected areas than in unprotected ones in the three MPAs analysed, as shown by the statistically significant effect of the fixed factor Location (Table 2, Fig. 3). In Cabrera and Cabo de Palos, the values of the analysed parameters in the NTZ and BZ areas were similar, and presented significantly higher values than in unprotected areas (Table 2, Fig. 3). In the case of Tabarca (both in rocky bottoms and *Posidonia* meadows) the NTZ showed greater values of the three parameters analysed compared to both BZ and UP areas (Fig. 3). A significant medium-scale (among sectors) variability was evidenced in Cabo de Palos, mostly in the unprotected locations, and a fine-scale (among zones) variability was detected for all dependent variables in Tabarca –rocky and Cabo de Palos (Table 1).

**Table 2:** Permutational multivariate analysis of variance (PERMANOVA) results for abundance, biomass and mean weight of *E. marginatus* found in the locations studied on Western Mediterranean Sea where groupers are present both inside and outside the MPA.

Marine Reserve			Abundance			Biomass			Mean Weight		
	Source	df	MS	PF	p	MS	PF	p	MS	PF	p
**CAB**	**L**	2	9960.3	14.43	**0.006**	29525	22.05	**0.001**	26915	23.99	**0.001**
	**S(L)**	6	660.0.	1.92	0.120	1278.3	1.13	0.374	1070.8	1.01	0.454
	**Z(S(L))**	18	344.9	1.53	0.073	1137	1.18	0.277	1065.5	1.16	0.310
	**Res**	128	224.6			965.7			922.0		
**TAB-r**	**L**	1	3174.3	92.15	**0.001**	21930	271.77	**0.0001**	22613	275.57	**0.0001**
	**S(L)**	4	28.8	0.10	0.981	67.4	0.035	0.998	68.6	0.003	0.998
	**Z(S(L))**	12	290	2.83	**0.002**	2032.2	2.97	**0.002**	2190.7	3.15	**0.0005**
	**Res**	83	102.3			682.2			695.8		
**CP**	**L**	2	2436.2	4.09	0.059	11668	5.03	**0.037**	11267	5.11	**0.030**
	**P** ***vs*** **UPs**	1	4816	6.83	**0.044**	22996	8.79	**0.020**	22204	8.97	**0.025**
	**UPs**	1	0.002	0.0005	0.984	3.4	0.008	0.984	3.4	0.008	0.984
	**S(L)**	6	623.6	3.35	**0.009**	2419.7	2.71	**0.027**	2298.1	2.66	**0.031**
	**S(P** ***vs*** **UPs)**	4	905.9	3.44	**0.031**	3327.9	2.67	0.067	3141.9	2.61	0.069
	**S(UPs)**	4	59.9	4.25	**0.023**	450.5	3.95	**0.017**	450.5	3.95	**0.021**
	**Z(S(L))**	18	186.7	1.45	0.108	895.8	1.69	**0.042**	868.4	1.69	**0.046**
	**Z(S(P** ***vs*** **UPs))**	12	270.5	2.20	**0.010**	1282.3	2.50	**0.006**	1241.3	2.49	**0.005**
	**Z(S(UPs))**	12	14	0.60	0.859	113.59	0.67	0.807	113.6	0.67	0.802
	**Res**	128	129.1			531.2			515.2		

L: locality, S: sector, Z: zone, P: protected, UP: unprotected, res: residuals, df: degree-of-freedom, MS: mean square, PF: pseudo-F, p: significance level (bold characters are used for significant results). CAB – Cabrera; TAB-r – Tabarca rocky habitat; CP – Cabo de Palos.

No significant effect of the factor Location was found in any MPA analysed for *E. costae* and *M. rubra* (Tables 3 and 4), despite these species were more abundant inside the protected area of Cabrera and Cabo de Palos (Fig. 3). This result was likely due to the high spatial variability at several scales, including among replicates. At Cabo de Palos a significant spatial variability among sectors within the protected location in abundance, biomass and mean weight of *E. costae* was evident (Table 3). Significant variability among zones within sectors was also found in all MPAs analyzed for *E. costae*, and in Tabarca – rocky and Cabo de Palos for *M. rubra* (Table 4).

**Table 3:** Permutational multivariate analysis of variance (PERMANOVA) results for abundance, biomass and mean weight of *E. costae* found in the locations studied on western Mediterranean Sea where groupers are present both inside and outside the MPA.

Marine Reserve			Abundance			Biomass			Mean Weight		
	Source	df	MS	PF	p	MS	PF	p	MS	PF	p
**CAB**	**L**	2	428.8	2.85	0.129	3034.1	3.52	0.082	3015.3	3.61	0.083
	**S(L)**	6	144.3	0.92	0.507	826.9	0.73	0.640	800.4	0.71	0.652
	**Z(S(L))**	18	157.6	2.12	**0.010**	1140.9	2.24	**0.005**	1133.4	2.26	**0.005**
	**Res**	135	74.4			509.0			501.6		
**TAB-r**	**L**	1	13.5	0.24	0.750	143.5	0.34	0.693	175	0.36	0.681
	**S(L)**	4	69.6	1.38	0.29	476.9	1.16	0.398	542.1	1.14	0.406
	**Z(S(L))**	12	52.5	2.18	**0.019**	428.7	2.59	**0.006**	495.9	2.68	**0.006**
	**Res**	83	24.1			165.8			185.3		
**CP**	**L**	2	170	0.26	0.798	1443.5	0.35	0.733	1444	0.36	0.732
	**P** ***vs*** **UPs**	1	40.3	0.05	0.869	363.31	0.07	0.832	352.8	0.07	0.826
	**UPs**	1	150.9	4.82	0.089	1257.4	4.89	0.084	1262.7	4.90	0.084
	**S(L)**	6	680.7	6.09	**0.0001**	4343.7	5.86	**0.0001**	4260.5	5.85	**0.0001**
	**S(P** ***vs*** **UPs)**	4	1041.5	7.87	**0.0001**	6643.4	7.54	**0.0001**	6516.9	7.53	**0.0001**
	**S(UPs)**	4	31	0.60	0.684	256.1	0.75	0.585	256.4	0.76	0.577
	**Z(S(L))**	18	112.4	1.80	**0.030**	744.2	1.69	**0.044**	731.8	1.68	**0.048**
	**Z(S(P** ***vs*** **UPs))**	12	136	2.16	**0.015**	904	2.03	**0.026**	888.1	2.02	**0.025**
	**Z(S(UPs))**	12	51.4	1.03	0.431	342.5	0.94	0.525	338.3	0.94	0.522
	**Res**	128	62.5			439.8			435.5		

L: locality, S: sector, Z: zone, P: protected, UP: unprotected, res: residuals, df: degree-of-freedom, MS: mean square, PF: pseudo-F, p: significance level (bold characters are used for significant results). CI – Cabrera; TAB-r – Tabarca rocky habitat; CP – Cabo de Palos.

**Table 4:** Permutational multivariate analysis of variance (PERMANOVA) results for abundance, biomass and mean weight of *M. rubra* found in the locations studied on Western Mediterranean Sea where groupers are present both inside and outside the MPA.

Marine Reserve			Abundance			Biomass			Mean Weight		
	Source	df	MS	PF	p	MS	PF	p	MS	PF	P
**CAB**	**L**	2	1.2	0.11	0.897	14.3	0.13	0.909	14.2	0.13	0.903
	**S(L)**	6	17.9	1.31	0.290	153.9	1.40	0.251	153.8	1.39	0.259
	**Z(S(L))**	18	13.6	0.83	0.685	110.0	0.83	0.685	109.9	0.82	0.683
	**Res**	135	16.3			131.8			131.8		
**TAB-r**	**L**	1	18.6	0.46	0.576	168.8	0.43	0.596	166	0.42	0.602
	**S(L)**	4	54.2	0.67	0.624	497.5	0.77	0.580	507	0.76	0.574
	**Z(S(L))**	12	83.9	2.17	**0.016**	674.9	2.27	**0.014**	689.4	2.30	**0.014**
	**Res**	83	38.6			297.1			299.2		
**CP**	**L**	2	44.2	1.35	0.325	441.9	1.32	0.330	441.9	1.32	0.341
	**P** ***vs*** **UPs**	1	87.3	2.06	0.218	873.1	2.03	0.208	873.1	2.03	0.214
	**UPs**	1	1.3	0.38	0.597	11.2	0.38	0.598	11.2	0.38	0.598
	**S(L)**	6	32.7	0.88	0.570	334.8	0.92	0.550	334.8	0.92	0.553
	**S(P** ***vs*** **UPs)**	4	46.6	0.91	0.544	481.0	0.95	0.521	481.3	0.95	0.535
	**S(UPs)**	4	3.6	0.57	0.744	29.6	0.57	0.736	29.6	0.57	0.746
	**Z(S(L))**	18	37.3	1.35	0.164	366.4	1.40	0.131	366.4	1.40	0.135
	**Z(S(P** ***vs*** **UPs))**	12	52.8	2.00	**0.031**	522.1	2.11	**0.021**	522.1	2.11	**0.017**
	**Z(S(UPs))**	12	6.3	1.15	0.327	52.3	1.15	0.331	52.3	1.15	0.324
	**Res**	128	27.8			261.9			261.9		

L: locality, S: sector, Z: zone, P: protected, UP: unprotected, res: residuals, df: degree-of-freedom, MS: mean square, PF: pseudo-F, p: significance level (bold characters are used for significant results). CI – Cabrera; TAB-r – Tabarca rocky habitat; CP – Cabo de Palos.

**Figure 4:**
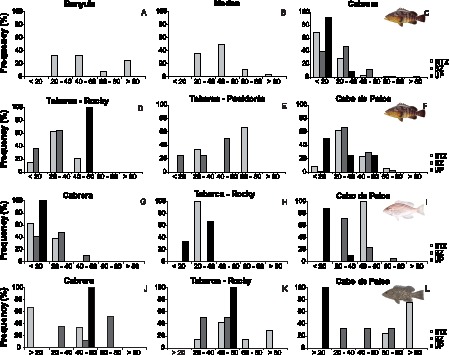
Distribution of frequencies of groupers size classes in each studied MPA (a–f, *E. marginatus*; g–i, *E. costae*; j–l, *M. rubra*).

The size structure of *E. marginatus* populations was more evenly distributed within Banyuls and Medes marine reserves, with the presence of large individuals, than inside the other MPAs, where individuals larger than 60 cm were scarce (Fig. 4). In all MPAs, larger individuals, if censused, were only seen within MPAs limits, while small ones were abundant outside MPA in Cabrera and Cabo de Palos (Fig. 4). Small individuals (<40 cm) of *E. costae* were also more frequently seen outside MPAs, while large individuals (>60 cm) of *M. rubra* occurred exclusively within MPA limits (Fig. 4).

### Influence of Habitat

Habitat structure appeared to exert a great influence on the abundance, biomass and mean individual weight of *E. marginatus* in Tabarca (both in rocky and *Posidonia* bottoms) and Cabo de Palos (Table 4). Abundance of *E. marginatus* in Banyuls and of *E. costae* in Cabrera, Tabarca rocky and Cabo de Palos was positively correlated to habitat structure. Habitat exerted also a significant influence on the mean weight of *E. costae* individuals in Tabarca – rocky and Cabo de Palos, and also on biomass of *M. rubra* in Cabo de Palos (Table 5). In the cases where it resulted to be significant, the influence of habitat on the parameters examined attained on average around 33% of total variability (range 14–53%) (Table 5).

**Table 5:** Summary of results of multiple linear regressions (adjusted *R* ^2^ and significance level) of mean abundance (Ab), biomass (Bm) and mean individual weight (IW) of the three species of groupers studied against linear, quadratic and cubic terms of all habitat characteristics measured in the transects for each MPA.

		Banyuls	Medes	Cabrera	Tabarca – rocky	Tabarca – *Posidonia*	Cabo de Palos
***E. marginatus***	**Ab**	0.210 (*)	0.117 (ns)	0.062 (ns)	0.296 (**)	0.525 (***)	0.331 (***)
	**Bm**	0.176 (ns)	0.048 (ns)	0.086 (ns)	0.350 (***)	0.473 (***)	0.359 (***)
	**IW**	0.083 (ns)	0.066 (ns)	0.112 (ns)	0.323 (**)	0.451 (***)	0.374 (***)
***E. costae***	**Ab**			0.139 (*)	0.257 (*)		0.253 (**)
	**Bm**			0.060 (ns)	0.173 (ns)		0.191 (ns)
	**IW**			0.084 (ns)	0.300 (**)		0.211 (*)
***M. rubra***	**Ab**			0.049 (ns)	0.397 (***)		0.210 (*)
	**Bm**			0.048 (ns)	0.366 (***)		0.159 (ns)
	**IW**			0.044 (ns)	0.378 (***)		0.159 (ns)

(ns: not significant; *: *p*<0.05; **: *p*<0.01; ***: *p*<0.001).

### Gradients of Biomass Export

Raw values of grouper biomass decreased with increasing distance from the boundary of MPAs, for Cabrera, Tabarca – rocky (except for *E. costae* in Tabarca – rocky), Cabo de Palos – North and Cabo de Palos – South, and these relationships were statistically significant in 8 out of the 11 species by MPA studied cases (Table 6). When residuals of biomass data obtained from multiple linear regressions were used as dependent variables instead of raw data, the number of significant negative correlations with distance decreased. Remarkably, the non-significant negative relationship of *E. costae* raw biomass southwards of Cabo de Palos became a significant positive relationship when using residuals (Table 6). These shifts when using residuals instead of raw data as dependent variables suggested a high habitat effect superimposed to that of protection measures.

**Table 6:** Results of linear correlations performed on raw data and residuals after extracting habitat effects on the biomass of the three studied grouper species (significant *p*-value in bold).

	*E. marginatus*				*E. costae*				*M. rubra*			
	Raw		Res		Raw		Res		Raw		Res	
	r	p	r	p	r	p	r	p	r	p	r	p
**Cabrera**	–0.483	**0.000**	–0.450	**0.000**	–0.140	**0.027**	–0.109	0.084	–0.056	0.374	–0.074	0.239
**Tabarca–rocky**	–0.413	**0.000**	–0.158	0.077	0.012	0.890	–0.071	0.429	–0.076	0.397	–0.021	0.814
**C. Palos North**	–0.516	**0.000**	–0.181	0.061	–0.216	**0.025**	0.047	0.627	–0.276	**0.004**	–0.057	0.556
**C. Palos South**	–0.573	**0.000**	–0.085	0.380	–0.156	0.107	0.226	**0.019**	–0.316	**0.001**	–0.121	0.212

**Figure 5:**
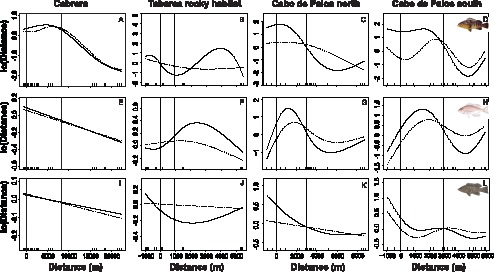
General additive model fitted for raw data (solid line) and residuals (dashed line) of biomass (Log_[x+1]_ transformed) of *E. marginatus, E. costae* and *M. rubra* as a function of the smooth variable distance (in m) from the boundaries of integral reserve. The y-axis is scaled so zero corresponds to the mean in log scale. The solid vertical line indicates the limit of the no-take zone (NTZ) and the dashed vertical line indicates the limit of the buffer zone (BZ).

Results of general additive models (GAM) on fish raw biomass as a function of distance to reserve boundaries yielded significant non-linear relationships in 8 out of 12 studied crossings between MPAs and species, these significant models explain 16–58% of total data variability (Table 7). When using residual biomass resulting from multiple linear regressions with habitat variables, the number of significant non-linear relationships with distance dropped to 5, and the range of percentage variability explained by the models decreased to 10–39% (Table 7). Graphical representation of GAMs evidenced three major patterns: the biomass of grouper species decreased i) constantly between integral reserve, buffer zone and outside (Fig. 5 b, c, g); ii) rapidly between the integral reserve and the buffer zone (Fig. 5 f, i, l); or iii) at the edge of the MPA, in the fishing area (Fig. 5 a, d, h, j, k). No difference was observed among raw and residuals data pattern for Cabrera, which was the expected pattern when habitat structure does not influence grouper abundances either inside or outside the MPA (Fig. 2). However, for the curves constructed with residuals of biomass data from Tabarca and Cabo de Palos, slopes were less pronounced and shapes smoother, following what it was hypothesized when habitat quality is better inside than outside the MPA. The actual shape of the observed biomass gradients suggested in these cases that there was no export, or it occurred at a very short distance (<1000 m).

**Table 7:** Analysis of deviance for the generalised additive models (GAMs) fitted with raw data and residuals of multiple linear regressions with habitat variables on biomass of the three studied grouper species.

		Term	Res df	F	p	R^2^
***E. marginatus***						
**Cabrera**	**Raw**	lo (distance)	246	3.73	0.005	0.28
	**Res**	lo (distance)	246	3.80	0.004	0.25
**Tabarca – rocky**	**Raw**	lo (distance)	120	6.87	<0.001	0.32
	**Res**	lo (distance)	120	1.48	0.211	0.07
**C Palos North**	**Raw**	lo (distance)	102	13.64	<0.001	0.54
	**Res**	lo (distance)	102	2.37	0.05	0.12
**C Palos South**	**Raw**	lo (distance)	102	12.00	<0.001	0.51
	**Res**	lo (distance)	102	3.25	0.02	0.10
***E. costae***						
**Cabrera**	**Raw**	lo (distance)	246	1.20	0.31	0.04
	**Res**	lo (distance)	246	1.11	0.35	0.03
**Tabarca – rocky**	**Raw**	lo (distance)	120	2.42	0.053	0.07
	**Res**	lo (distance)	120	1.15	0.33	0.03
**C Palos North**	**Raw**	lo (distance)	102	29.12	<0.001	0.58
	**Res**	lo (distance)	102	14.68	<0.001	0.39
**C Palos South**	**Raw**	lo (distance)	102	20.61	<0.001	0.40
	**Res**	lo (distance)	102	10.97	<0.001	0.29
***M. rubra***						
**Cabrera**	**Raw**	lo (distance)	246	1.55	0.18	0.03
	**Res**	lo (distance)	246	1.35	0.25	0.03
**Tabarca – rocky**	**Raw**	lo (distance)	120	2.09	0.08	0.07
	**Res**	lo (distance)	120	0.70	0.59	0.02
**C Palos North**	**Raw**	lo (distance)	102	2.37	0.05	0.16
	**Res**	lo (distance)	102	1.47	0.21	0.06
**C Palos South**	**Raw**	lo (distance)	102	2.37	0.05	0.16
	**Res**	lo (distance)	102	1.47	0.21	0.06

## Discussion

### Reserve Effect on Grouper Populations

In this work, we showed that grouper species, and especially *E. marginatus*, are extremely favoured by protection measures in Western Mediterranean MPAs. Average abundance, biomass and individual weight of groupers are higher within the protected areas than immediately outside. In some MPAs (Banyuls, Medes, Tabarca – *Posidonia*), dusky groupers were observed only inside the MPA, indicating the occurrence of a high fishing effort just at the edge of the protected areas [17], [38]. Therefore, at present abundant grouper populations (≥1 indiv.125 m^−2^) are found only within MPAs in the western Mediterranean, highlighting the heavy impact of fishing on the coastal fish populations and the importance of MPAs to maintain epinephelid populations. A noticeable recovery in grouper abundance as a response to fisheries closure has been already documented in the Mediterranean Sea [39]–[41], [24], corroborating that these species respond more or less rapidly to protection [4], [28], [42] even in small reserves [29]. Thus, MPAs are very effective to counteract the rapid depletion of predatory fish observed worldwide [43]. Because high biomass of top predators can be considered the natural state of marine reef fish communities, as demonstrated by studying remote reefs [26], [44], MPAs would serve to recover pristine fish community structures [25]. The key questions are how much protection time is required until local carrying capacity is attained [42], and which is the maximum value of biomass of apex predator [26] for unfished Mediterranean locations?

Regarding individual size of groupers, almost all size classes are represented within the studied MPA, with larger individuals generally restricted to inside MPA limits. This indicates that grouper populations protected from fisheries are well established and are constituted from both mature and juvenile individuals. The high abundance, large size and stable size class distribution can allow the reproduction of groupers inside MPAs [45]–[47]. Due to the high site fidelity and strong territorial behaviour displayed by mature dusky grouper [48], younger individuals must swim larger distances in order to find food, shelter and constitute their own territory. During this wandering search, juveniles may establish themselves outside MPAs as a density-dependent response to high competition for territory inside the reserve [49]. Both mechanisms, egg and larvae exportation from the restored spawning biomass and density-dependent movement of juveniles, can be reflected by an increased observation of younger dusky grouper outside the limits of marine reserves [50].

Our study shows important geographical differences in species composition, abundance and size structure of grouper populations. Grouper species were generally more frequent and abundant in the southernmost MPAs (Cabo de Palos, Tabarca and Cabrera), where the three species were found. Moreover, in the northernmost reserves, only dusky groupers (in Banyuls and Medes) or even no groupers at all (in Carry) were recorded. Dusky groupers showed also much lower abundances in northern MPAs than in southern ones. Additionally, the higher mean biomass and individual weight values recorded for this species in Banyuls and Medes indicated that *E. marginatus* populations were composed of larger and older individuals than in the other MPAs, which could be due to the older ages of these two marine reserves (dating from 1974 and 1983, respectively), compared to the southernmost MPAs (from 1986 for Tabarca to 1995 for Cabo de Palos). On the other hand, environmental conditions could also shed light on the spatial differences observed. Duration of the spawning activity and the spawning survivorship (recruitment success) are limited by temperature thresholds, and can largely influence the differential population parameters observed [46]–[47]. The geographic pattern observed in this study corroborates the thermal affinity of grouper species, especially *E. costae* and *M. rubra*. Moreover, in our data juveniles were virtually absent from the northern MPAs despite the recovery of the adult population and years of protection, although this pattern could be reverting during the last decade due to seawater warming [50]. Thus, latitudinal/oceanographic effects could be limiting northern populations.

### Biomass Export of Groupers

Identifying life-stages that are critical to the population dynamics of a threatened species along its distribution range is essential to effectively design MPA networks in order to achieve conservation objectives. Based on the estimation of biomass gradients, significant evidences of spillover of groupers from inside to outside MPA borders taking the effect of habitat into account were detected in only two of the five marine reserves analyzed, Cabo de Palos and Cabrera. In Cabo de Palos, an area with low artisanal fishing effort directed to grouper species [17], the spatial scale of grouper spillover seemed to be of less than 1000 m, a value lower than that estimated by Harmelin-Vivien et al. [16] for the whole visually-censused fish assemblage (3000 m). This observation corroborates the hypothesis that biomass gradients are sharper (and thus spillover lower) for low-mobility or high-catchability species, provided that fishing pressure outside the MPA is high compared to that exerted in the BZ, as it is clearly the case in Cabo de Palos [17], [38]. It is important to highlight that groupers are targeted by recreational fishing (mainly spearfishing) as heavily as by professional fishing [51], and spearfishing is highly practiced around the Cabo de Palos marine reserve (Carlos W. Hackradt, *personal observation*). In Cabrera, the closer unprotected sites are located at about 10 km from the MPA with unsuitable habitats for juvenile groupers in between. Therefore, grouper biomass export from Cabrera MPA is rather likely due to egg and larval dispersal, as shown by Crec’hriou et al. [11]. In Banyuls, Medes and Tabarca, no spillover of groupers outside the reserves could be detected. In Cabo de Palos and Cabrera, grouper abundances were the highest among the six MPAs studied, and there was no difference in grouper abundance and biomass between NTZs and BZs, where artisanal fishing occurred, while in Tabarca higher abundances were recorded in the NTZ than in the BZ. We hypothesise that the importance of biomass gradients (and thus the occurrence of fish spillover) is likely to be a function of fish density inside MPAs [20]: when groupers are abundant inside MPA, biomass gradients from inside to outside are to be evidenced, as a result of movement of individuals (usually small-sized ones); when, in turn, there is a low number of individuals within an MPA, gradients could be detected from the NTZ to the BZ, but not from the BZ to outside the MPA. Therefore, because the efficiency of an MPA for a target species will depend on its total size compared to the home range of this species [21], the carrying capacity, zonation and size of the MPA should be taken into consideration when estimating its potential for spillover. Although several studies establish that Mediterranean groupers have high site fidelity and small home range [48], [52], further studies about the mobility and home range of these species inside and outside Mediterranean MPAs are still needed, taking into account fish densities and sizes.

### Habitat Characteristics and MPA Designs

The present study emphasizes the importance of considering explicitly habitat structure when evaluating biomass exportation patterns [14], [16], [24]. The influence of habitat structure on the shape of the curve depicting the relationship between the response variable and the geographical distance to MPA limits tells much about the relative importance of structural habitat to favour or, at contrary, to hinder spillover. In those MPAs where habitat structure appears to exert a great influence on grouper abundance, biomass and mean individual weight (Tabarca – rocky and Cabo de Palos), extracting the habitat effect smoothes the shape of biomass gradient, although it does not affect the estimated spillover distance. According to the theoretical model proposed here, this shift is likely due to the fact that habitat structure is of higher quality within than outside MPAs, which is a quite normal situation in the Mediterranean [24]. In these situations, structural habitat, by providing additional food and refuge resources, would act as an “attractant” for groupers, hence boosting the carrying capacity of protected sites and, consequently, reducing the strength of spillover to neighbouring, unprotected sites. However, Cabo de Palos – Islas Hormigas marine reserve can be considered a singularity within the Mediterranean context regarding the habitat structure: the offshore steep and structurally complex rocky shoals, with significant water motion and currents, are not typical of most Mediterranean rocky reefs [53]. The fact that, despite these particular features, some spillover can be detected highlights that this process is possible even for highly sedentary fish species, once a minimum density has been attained. Moreover, spillover is likely to be favoured by the existence of habitat continuity from inside to outside the MPA [17], [38], [54]. In this work spillover has not been detected when exploring continuous habitats (*Posidonia oceanica* meadows), contrarily to what has been observed in discontinuous (rocky bottoms) habitats around Tabarca MPA. On the other hand Forcada et al. [54] working in the same location, observed decreasing total abundance gradients in both habitats studied, *P. oceanica* meadow and rocky substrate, and independently of their different continuity through the reserve boundaries. Although groupers can be observed in seagrass meadows, they primarily live in rocky bottoms, which could explain the fact that no groupers were observed in *P. oceanica* outside the reserve, and therefore the absence of spillover in continuous habitats.

Further from oceanographic and hydrological factors (see above), low recruitment level of groupers in northernmost locations could be explained in part by the specific habitat features existing in each MPA, given that spatial variability in reef fish recruitment at the scale of locations (thousands of metres apart) as well as sites (separated by hundreds of metres) are likely a reflection of habitat differences at these spatial scales [55]. Juvenile groupers show a preference for cavities and recesses in shallow areas from near-surface bottoms to 10 m depth [56]. Therefore, the capacity of marine reserves to protect early fish life stages will rely on the extent to which these crucial habitat characteristics are included within the protected areas [57].

These results provide novel insights into the debate about the consequences of establishing a buffer zone when designing an MPA. Some studies argue that BZ’s can have detrimental effects on the protection of fish species [3], and that only no-take marine reserves should be created, as partial protection is an ineffective conservation strategy [58]. The present study found that the success of BZ to protect grouper species will depend on both the ecological and management conditions established in each particular MPA. The ecological role of a partially closed area to protect commercially important fish species would depend on a variety of aspects, such as the difference in habitat quality between both zones, the heterogeneity and spatial continuity of structural habitat between both areas of different protection levels and with unprotected ones, the intensity of fishing pressure allowed in the buffer zone compared to neighbouring unprotected areas, the relative size of both no-take and buffer zones, and the mobility and home range of the species involved [3]–[4]. On the other hand, it has been demonstrated that partial protection of coastal areas together with an adaptive co-management plan that involves fishers, scientists, and managers may benefit fishing communities and reduce overfishing [59].

In summary, the evidence presented here shows that MPAs are an essential tool to protect overexploited populations and threatened species, such as Mediterranean groupers, provided that they are adequately enforced and managed [10], [58], [60]. Moreover, MPAs are able to export fish biomass to neighbouring areas, even in the case of very sedentary species, but only if they are appropriately designed in terms of reserve location, size, zoning, and management. Therefore, spillover by eggs release and larval dispersion could be the key process to ensure the connectivity among distant sites for sedentary and strongly habitat-linked species such as groupers. Further studies on grouper population mobility, connectivity, habitat preferences, demography and carrying capacity are urgently needed in order to establish ecological criteria to optimize MPA design.
